# The traditional Chinese medicine *Achyranthes bidentata* and our *de novo* conception of its metastatic chemoprevention: from phytochemistry to pharmacology

**DOI:** 10.1038/s41598-017-02054-y

**Published:** 2017-06-20

**Authors:** Zhou Jiang, Jun Qian, Haiyan Dong, Jingyi Yang, Xiaobo Yu, Jianzhong Chen, Hongning Chen, Qing Shi, Lee Jia

**Affiliations:** 1 Cancer Metastasis Alert and Prevention Center, and Pharmaceutical Photocatalysis of State Key Laboratory of Photocatalysis on Energy and Environment, College of Chemistry; Fujian Provincial Key Laboratory of Cancer Metastasis Chemoprevention and Chemotherapy, Fuzhou University, Fuzhou, 350002 China; 2 Rutgers, The State University of New Jersey, 160 Frelinghuysen Road, Piscataway, NJ 08854-8020 USA

## Abstract

Our recent biosystems analysis revealed similarities between embryonic implantation and cancer cell adhesion, which suggests that abortifacients may be good for safe and effective metastatic chemoprevention targeting circulating tumor cells (CTC). Here we test the hypothesis by using the well-known abortion herb *Achyranthes bidentata Blume* (*A. bidentata*). Five compounds were separated from the herb root. Among them, ginsenoside Ro was the most potent in inhibiting embryonic implantation within non-cytotoxic concentrations. It specifically inhibited the metastatic dissemination capability of colon cancer cells HT29, including the migration and invasion ability, and their adhesion to human endothelium through inhibiting integrin *αvβ*6, MMP-2, MMP-9, and ERK phosphorylation by HT29. Pretreatment of nude mice with oral ginsenoside Ro followed by HT29 intravenous inoculation and 40-day oral ginsenoside Ro significantly prevented lung metastasis with downregulation of integrin *αvβ*6 and no toxicity. The present study firstly introduces the new conception of utilizing safe and effective abortion botanic medicines for CTC-based metastatic chemoprevention.

## Introduction

Recent cancer statistics reported that the number of cancer survivors after primary treatments will increase to 19 million by 2024 in the USA alone^1^. Although the good news signifies the success of today’s cancer primary treatments, it also underlines the urgent need for safe and effective cancer metastasis prevention strategies to protect those survivors from the deadly cancer recurrence and metastasis, which are hardly cured and responsible for 90% of cancer deaths. The root cause of cancer metastasis is considered circulating tumor cells (CTCs). However, currently there is neither specific treatment nor pre-metastatic chemoprevention targeting CTC-based metastasis.

In our searching for novel therapies targeting CTC-based metastasis, we first characterized colorectal patients’ CTCs as Ep-CAM^+^CD45^−^pancytokeratin^+^ cell population^2^. We then revealed that these CTCs usually possess more than one type of surface biomarkers. The discovery led us to engineering nanomaterials that can carry more than one type of antibodies or aptamers to target two surface biomarkers on one CTC simultaneously to enhance the specificity of recognizing and capturing the rare CTCs in patient blood^3–6^. The activity of the captured CTCs was shown suppressed. Furthermore, our understanding of CTC-based cancer metastatic tissue tropism helped us create a quardruple-combined drug called HAMPT (abbreviated for Highly Active Metastasis Preventing Therapy), which not only targets various CTC-based metastatic pathways, but also enhances patient’s own recovery power to suppress metastatic potential^7^.

Recently, after analyzing the molecular and cellular similarities and differences between embryonic implantation to uterine endometrium and CTCs adhesion to vascular endothelium, we found that many molecules, including integrins, cellular adhesion molecules (CAM) such as Ep-CAM, I-CAM, V-CAM, as well as selectin, hormone receptors, Sialyl lewis X, and MMPs, are shared by both the embryonic implantation system and cancer cell adhesion-invasion system. The analysis first inspired us to use the blockbuster abortifacient mifepristone (RU486) and its metabolite metapristone to test our hypothesis that abortifacients may be a class of safe and effective cancer metastatic chemopreventives. We also screened the abortion plants or herbs from the traditional Chinese medicine (TCM) for their potential metastatic chemopreventive effects. In the huge TCM treasure, we only focused on those phytomedicines that can meet the following criteria: good safety profile without cytotoxicity at chemopreventive doses; anti-adhesion (anti-implantation), anti-inflammation, anti-coagulation, analgesic, and vasodilation. The TCM *Murraya paniculata* (L.) Jack meets the above criteria. Both its crude extract and the compound isolated from the extract seem to be very promising as the pre-metastatic chemopreventives^8, 9^. As we continue to develop these components into the pre-metastatic chemopreventives, another herbal medicine caught our great attention.

*A. bidentata* and *Achyranthes aspera* Linn (*A. aspera*) belong to the botanic family Amaranthaceae. They can be found all over in Asian countries like India, Korea, Japan, and China. *A bidentata* was recorded in “Shen nong Ben cao Jing” (Shen nong’s Herbal), one of the world’s earliest pharmacopoeia, which was supplementary edited by Tao Hongjing during the Chinese Liang Dynasty (505–557 A.D.)^10^. The book briefly descripted *A. bidentata* and its abortion effectiveness. The ancient Chinese folk doctors placed the juice from the smashed root of *A. bidentata* into vagina to induce abortion, and the decoction from *A. bidentata* was used for female blood clots based on its anticoagulative activity. Today, the root of *A. bidentata* and its medical applications are prescribed and updated in the Chinese Pharmacopeia (2010 edition) as an important herbal medicine. Its multiple pharmacological effects include anti-osteoporosis^11, 12^, neurotrophic and neuroprotective effects^13, 14^, inhibition of myocardial ischemic/reperfusion-induced injury^15, 16^, antitumor and immunomodulatory activities^17–19^.

Previous phytochemical studies with *A. bidentata* discovered many active components such as phytosterone and phytoecdysteroids^20^, saccharides and saponins^21^, and others from the herb. Because of its important medicinal values, China recently completed geographic investigation on *A. bidentata* pollution-free distribution^22^. In India, plant tissue culture techniques have also been developed for *in vitro* callus production and direct green herbal regeneration of both *A. bidentata* and *A. aspera* using nodal segments^23^. The ubiquitous geographic distribution and abundant growth of *A. bidentata*, as well as its *in vitro* reproduction to meet the future pharmaceutical demand for its active components, *A. bidentata* seems to be the best TCM candidate for development into an affordable cancer metastatic chemopreventive if we also consider its safety profile and multiple beneficial pharmacological effects as we summarized above. Hence, we started de novo a project 3 years ago to search for active components from the TCM with the hope that the identified components meet the criteria of cancer metastatic chemopreventives. The new discovery is reported here for the first time.

## Results

### Fast bioactive component screening from raw root to isolate pure compounds

**Figure 1:**
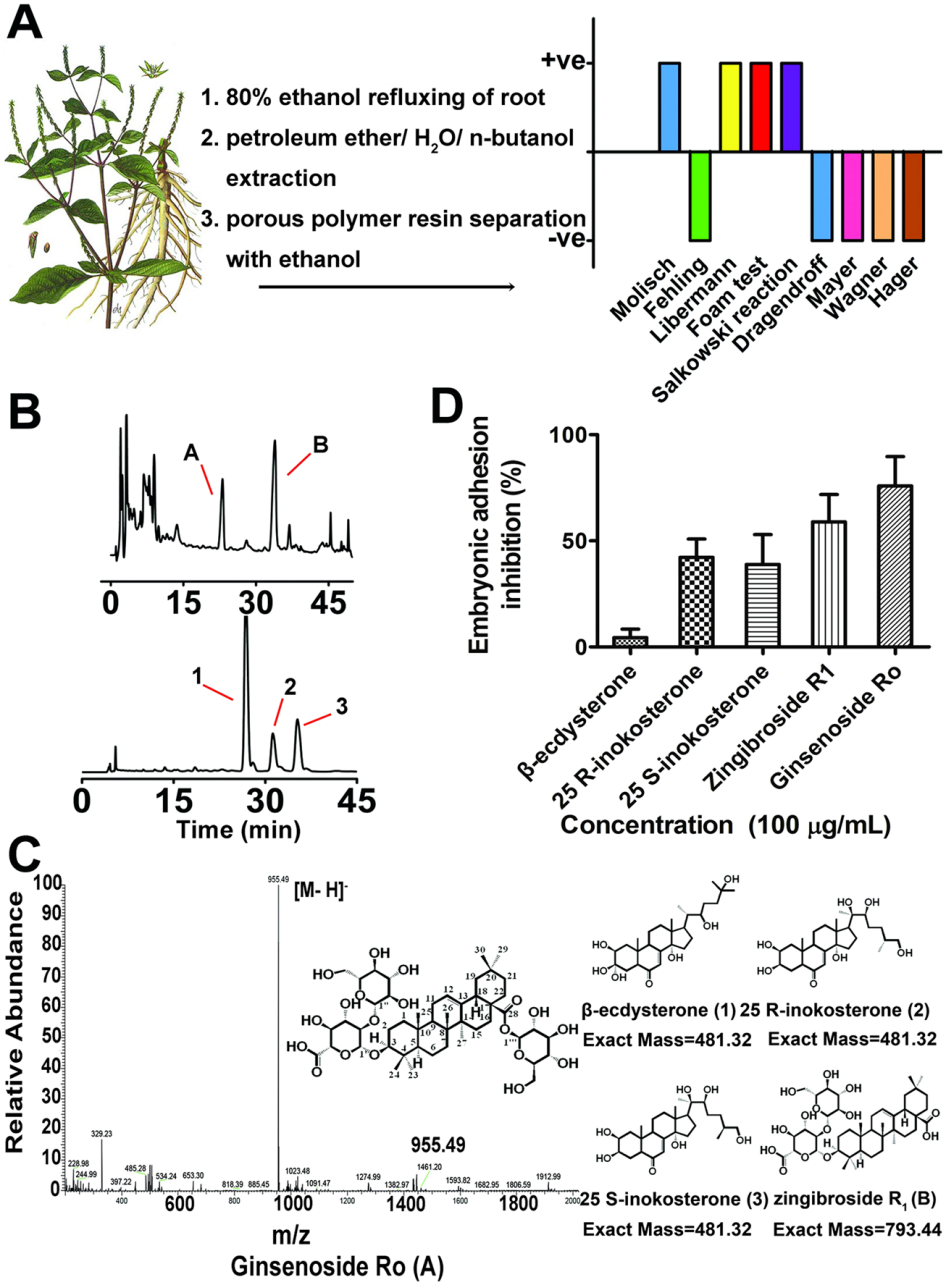
Phytochemical analysis and embryonic implantation comparison of five main components separated from *A. bidentata* root. (**A**) The smashed herb root was refluxed with 80% ethanol, and the concentrated residual was extracted with ether/water and then butyl alcohol, followed by resin column separation. The elute fraction was subjected to the standardized phytochemical screen that showed steroids positive by Salkowski and Lieberman-Burchardt assays; carbohydrates negative by Fehling’s test; glycosides positive by Molisch’s test; saponins positive by Lieberman, and foam assays; alkaloids negative by Dragendroff, Mayer, Wagner and Hager assays. (**B**) Further fine HPLC separation showed that the elution mainly contained two saponins and three sterones. (**C**) Mass and NMR analyses suggested that they are A: ginsenoside Ro; B: zingibroside R1; 1: *β*-ecdysterone; 2: 25 R-inokosterone; and 3: 25 S-inokosterone. (**D**) Human embryo/ endometrium adhesion test showed that ginsenoside Ro was the most potent among the five components in inhibiting the adhesion.

Our fast bioactive component screening started from the smashed root of *A. bidentata* to the crude extracts. Each extract obtained from different solvents was first subjected to bioactive screening using related molecular and cellular assays followed by the standardized phytochemical screening applied to the most interesting extract (Fig. 1A), and then the separation and characterization of the most active compounds from the most interesting extract. The fast bioactive screen procedure usually takes us 5–6 months to find the interesting compound(s)^8^. Briefly, the smashed root of *A. bidentata* was refluxed overnight with 80% ethanol. The concentrated fractions obtained from different solvent extracts were first screened by cell bioassay^8, 9^. Following the bioassay, we identified the *n*-BuOH-soluble fraction obtained after petroleum ether partition to be the most potential. It was then subjected to porous polymer resin D101 chromatography and yielded crude elution. By using semi-preparative HPLC under different eluting conditions (see Methods), a series of structurally related sterones and saponions were isolated and then purified to be five compounds (Fig. 1B). The structures of these compounds were elucidated based on their mass spectrometric analysis as *β*-ecdysterone (1), 25 *R*-inokosterone (2), 25 *S*-inokosterone (3), zingibroside R_1_ (B) (Fig. S1 and S2) and ginsenoside Ro (A) (Fig. 1C), respectively. The structures of these compounds were further verified by nuclear magnetic resonance (NMR). The pseudo-molecular ion of A was m/z 955.4912 [M − H]^−^ under the ESI-MS negative ion mode, which corresponds to a molecular formula of C_48_H_75_O_19_. The ^1^H-NMR (500 MHz, in C_5_D_5_N) resonance at δ(H) 1.25, 1.23, 1.08, 1.06, 0.88, 0.86, 0.81(s, 3 H, seven methyl groups), 3.16 (d, 1 H, H-18), 3.26 (dd, 1 H, H-3), 4.99 (d, 1 H, H-1′), 5.38 (s, 1 H, H-12, an olefinic proton), 6.29 (d, 1 H, H-1 of C-28aglycon). The detailed ^13^C-NMR data were shown in Supplementary Table S1. The above analysis gives a structure to be 3-*O*-[*β*-D-glucopyranosyl (l → 2) -*β*-D*-*glucopyranosyl] oleanolic acid 28- *O*-*β*-D-glucopyranosyl ester.

Meanwhile, high throughput spheroid attachment assay was adopted to robustly screen the 5 compounds for the best candidate that is the most potent in inhibiting embryonic implantation to human endometrium. Trophoblast spheroid formed spontaneously following cell aggregation was added to the confluent monolayer of human endometrial cells RL95-2 in the presence of compound 1, 2, 3, A and B, respectively, to test their individual ability of inhibiting the implantation. After twenty-four hour treatment, ginsenoside Ro appeared to be the most potent in inhibiting the implantation between embryo JAG-3 (human choriocarcinoma cell) and endometrial RL95-2 based on the number of attached spheroids (Fig. 1D). Hence, the following studies were focused on ginsenoside Ro.

### Effect of ginsenoside Ro on implantation of embryo to endometrium

**Figure 2:**
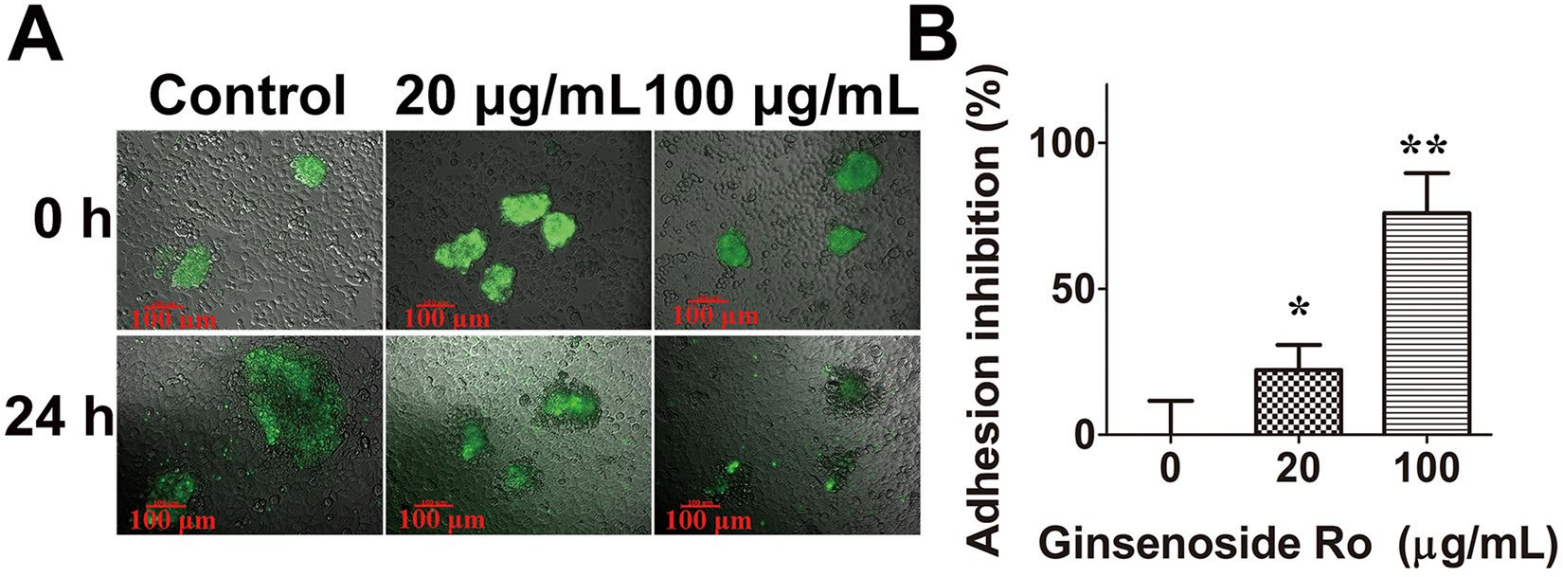
Inhibition of biomimetic human embryo implantation to endometrium by ginsenoside Ro. (**A**) Laser confocal microscopy imaging showed the significant inhibition by ginsenoside Ro of implantation of human spheroid embryo JEG-3 (green) to confluent monolayered human endometrial cells RL95-2 after 24 h co-incubation. (**B**) Concentration–dependent inhibition by ginsenoside Ro of spheroid embryo outgrowth of JEG-3 cells to human endometrial RL95-2 cells. Each bar represents the mean ± SD (n = 3).

To generate spheroids of JAG-3 cells for use as blastocyst model, the JAG-3 suspension was cultured with shaking at 70 rpm for 24 h until spheroids of 50–150 *µ*m in diameter (similar in size to an implanting blastocyst) were formed. The JAG-3 spheroids were then co-incubated with confluent endometrial RL95-2 cells in the presence and absence of ginsenoside Ro for 24 h (Fig. 2A). In the control implantation assay, co-culture of JAG-3 spheroids with endometrial cells resulted in adhesion of JAG-3 into the endometrial monolayer. In comparison with the control, ginsenoside Ro produced concentration-dependent inhibition of the adhesion (Fig. 2B). Additionally, embryonic outgrowth induced by endometrial epithelial layer was inhibited by ginsenoside Ro as shown in Fig. 2A that the JAG-3 spheroid outgrowth area (shaded area) was significantly smaller than that in control group. The result, for the first time, demonstrated at the cellular level that the compound isolated from the abortion TCM significantly inhibited embryonic implantation into endometrium.

### Low cytotoxicity of ginsenoside Ro

**Figure 3:**
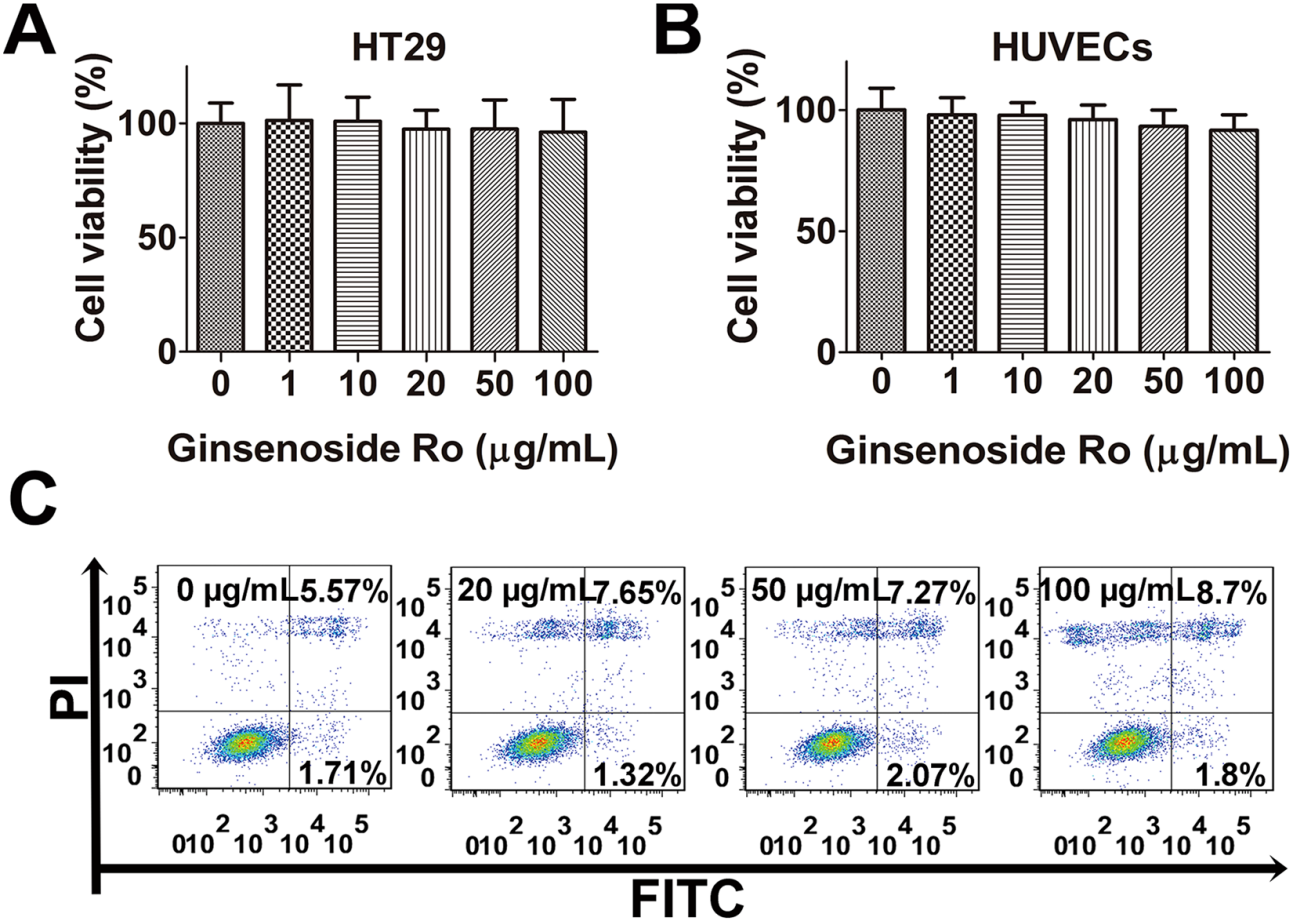
Effects of ginsenoside Ro on cell viability and proliferation. (**A**) and (**B**) Ginsenoside Ro within 100 *μ*g/mL produced no significant cytotoxicity against human colon cancer HT29 cells and endothelial HUVECs. (**C**) Flow cytometric analysis showed no significant apoptotic signs in the HT29 cells treated with ginsenoside Ro.

Cell viability assay revealed that ginsenoside Ro up to 100 μg/mL did not produce significant inhibition on HT29 viability after 24-h treatment (Fig. 3A). Ginsenoside Ro did not significantly inhibit viability of HUVECs, either (Fig. 3B). Fluorescence microscopic analysis showed no significant morphological changes in the nucleolus, internal organelle and plasma membrane integrity in the presence of ginsenoside Ro (100 *μ*g/mL). HT29 cells labeled with Annexin V and PI showed primarily Annexin V and PI negative, indicating that they were viable and did not undergo apoptosis after ginsenoside Ro treatment. Quantitative analysis r (Fig. 3C) showed a slight increase in the percentage of apoptotic population of HT29 exhibited in flow cytometric Q2 plot quadrant (up right) in comparison with the untreated control without significantly affecting the cell proliferation.

### Inhibition by ginsenoside Ro of adhesion, migration and invasion of HT29 cells

**Figure 4:**
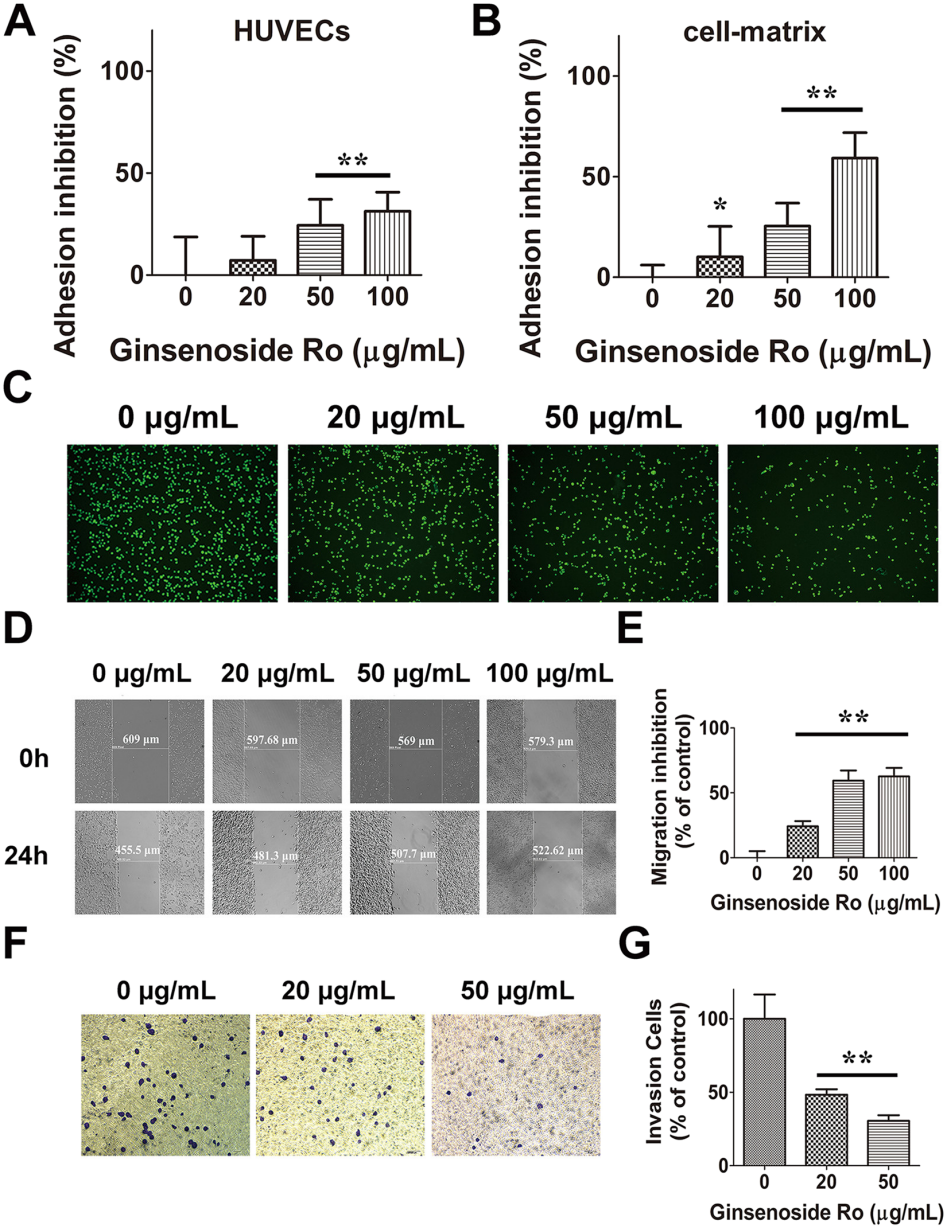
Ginsenoside Ro inhibited adhesion, migration and invasion of HT29 cells. (**A** and **B**) Ginsenoside Ro produced concentration-dependent inhibition of adhesion of HT29 cells to HUVECs (**A**), and matrigel (**B**); (**C**) Representative microscopic imaging showed concentration–dependent inhibition by ginsenoside Ro of adhesion of Rhodamine 123-labeled HT29 to HUVECs monolayers stimulated by IL-1*β* (1 ng/mL). (**D**) and (**E**). Inhibition by ginsenoside Ro of HT29 mobility following the scratch assay: (**D**) inserted microscopic images taken at 0 and 24 h after the scratches; (**E**) quantitative analysis of concentration-dependent inhibition by ginsenoside Ro of HT29 migration on fibronectin. (**F**) Representative images (magnification ×200) showing that HT29 cells (blue) passed through the transwell monolayer, and the cell’s invasion ability was inhibited by ginsenoside Ro. (**G**) Quantitative analysis of the concentration-dependent inhibition by ginsenoside Ro of the HT29 invasion ability. The number of cells passing through the transwell monolayer was counted in five separate microscopic fields. Data represent the mean ± SD. (n = 3–5); **P < 0.01, compared with the untreated control.

Cancer cell adhesion to extracellular matrix (ECM) is important for tumor invasion since it is a crucial step in proteinase-dependent cell locomotion. We first performed cell-matrix adhesion assay to investigate whether ginsenoside Ro inhibited adhesion of HT29 to matrix, where Basement Membrane Matrix (Matrigel^TM^, BD) used as the artificial ECM. Ginsenoside Ro at 50 and 100 µg/mL significantly inhibited adhesion of HT29 to matrigel by 29.2 ± 8.1% and 61.5 ± 8.3%, respectively (*P* < 0.01; Fig. 4B). In addition, adhesion of HT29 labelled with fluorescent rhodamine 123 to HUVECs was inhibited by ginsenoside Ro in a concentration-dependent manner. For instance, ginsenoside Ro at 50 and 100 μg/mL produced inhibition by 28.7 ± 5.4% and 37.3 ± 3.9%, respectively, of the cell-cell adhesion (*P* < 0.01) compared with that of the untreated control group (Fig. 4A). Fluorescent microscopic observation at 507 nm excitation wavelength showed that adhesion of HT29 to HUVEC monolayer was reduced by ginsenoside Ro in a concentration-dependent manner (Fig. 4C).

Migration and invasion are the key steps of the metastatic cascade. Based on the above cell-matrix adhesion result, we then further investigated the effect of ginsenoside Ro on motility of HT29 cells on the fibronectin, using the established protocol^24^. As shown in Fig. 4D, the migration rate of HT29 cells was qualified by scratch width in a confluent monolayer on the fibronectin. After treatment of HT29 with ginsenoside Ro for 24 h, the directional motility or spreading rate of HT29 was significantly inhibited in the presence ginsenoside Ro at 50 and 100 *μ*g/mL, compared with the control group. The representative images and quantitative data are shown in Fig. 4D and E, respectively.

To study the influence of ginsenoside Ro on the invasive ability of HT29 cells, we performed the transwell invasion assay. As observed under microscopy (X 200; Fig. 4F), treatment of HT29 labeled in blue with ginsenoside Ro reduced ability of the cells infiltrating the Matrigel membrane. The invasion ability of HT29 was reduced by 52% and 73% by 20 and 50 *μ*g/mL ginsenoside Ro, respectively (*P* < 0.01; Fig. 4G). In short, ginsenoside Ro reduced the motility and invasive capability of HT29.

### Ginsenoside Ro targets integrin *αvβ*6-associated ERK pathway and inhibits MMP secretion

**Figure 5:**
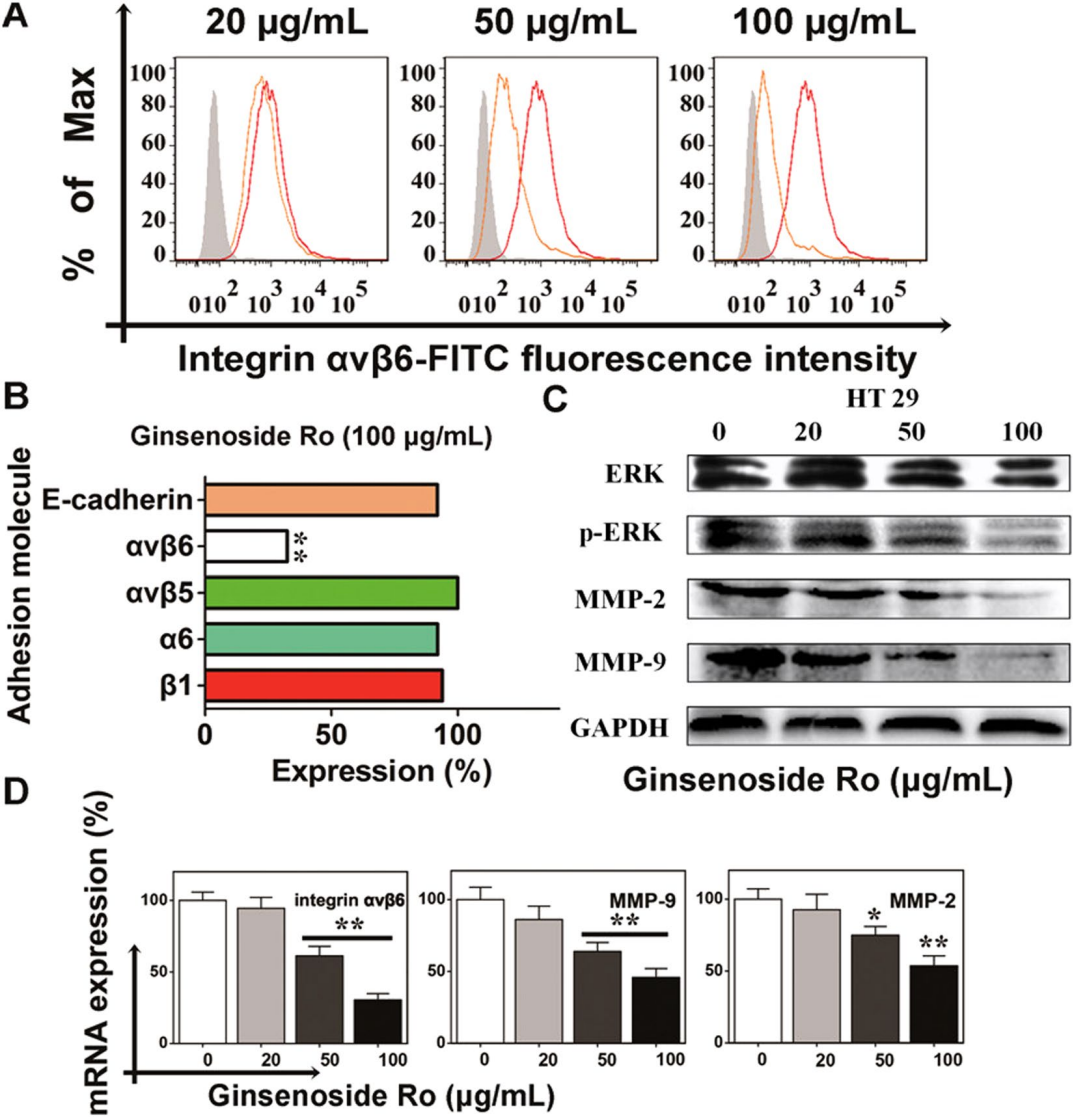
Ginsenoside Ro interfered with integrin *α*v*β*6-associated ERK pathway in HT29. (**A**) Flow cytometric imaging showed expression of integrin *α*v*β*6 on HT29 cells and the inhibition by ginsenoside Ro of the expression. (**B**) Flow cytometric analysis showed that ginsenoside Ro (50 *µ*g/mL) specifically inhibited integrin *α*v*β*6 expression in HT29 without affecting other integrins and E-cadherin. (**C**) Western blotting stains showed that ginsenoside Ro inhibited expression by HT29 of MMP-2, MMP-9 and ERK phosphorylation in a concentration-dependent manner, and (**D**) the inhibition was further supported by the real-time PCR analysis measuring the transcript levels of integrin *α*v*β*6, MMP-2 and -9 in HT29 cells. Each bar represents the mean ± SD, (n = 3–5). *P < 0.05, and **P < 0.01, compared with the control.

To explore the mechanisms by which ginsenoside Ro inhibits adhesion, motility, and invasion of cancer cell HT29, we first utilized flow cytometry to analyze changes in expression of various cellular adhesion molecules (CAMs) such as integrins *αvβ*5, *αvβ*6, *α*6, *β*1 and E-cadherin in the presence and absence of ginsenoside Ro. These CAMs are often found involved in migration and invasion of tumor cells as well as implantation of embryos. The flow cytometric images showed clearly that ginsenoside Ro significantly inhibited expression of integrin *αvβ*6 (Fig. 5A) with no appreciable effect on expression of other integrins and E-cadherin (Fig. 5B).

Given that integrin *αvβ*6, matrix metalloproteinase (MMP)-2 and -9 are responsible for cell invasion and cell-matrix adhesion and that the *β*6- extracellular signal-regulated kinase 1/2 (ERK1/2) interaction mediates MMP-2 and -9 secretion^25–27^, we further studied if ginsenoside Ro suppresses the expression of integrin *αvβ*6, MMP-2 and MMP-9 at the protein and mRNA levels by using western blotting and qRT-PCR methods. As shown in Fig. 5C, ginsenoside Ro at 100 μg/mL significantly inhibited the ERK phosphorylation, and expression of MMP-2 and MMP-9 proteins. To verify the above observation, we also ran qRT-PCR analysis, which showed that mRNA expression of integrin *αvβ*6, MMP-2 and MMP-9 was down-regulated to 30, 47 and 55% of the control, respectively (*P* < 0.01), by 100 *μ*g/mL of ginsenoside Ro. These results indicate that ginsenoside Ro inhibits adhesion, migration and invasion of HT29 cells by inhibiting the expression of MMP-2, -9, integrin *αvβ*6, and p-ERK1/2.

### Ginsenoside Ro inhibits metastasis of HT29 cells to mouse lungs

**Figure 6:**
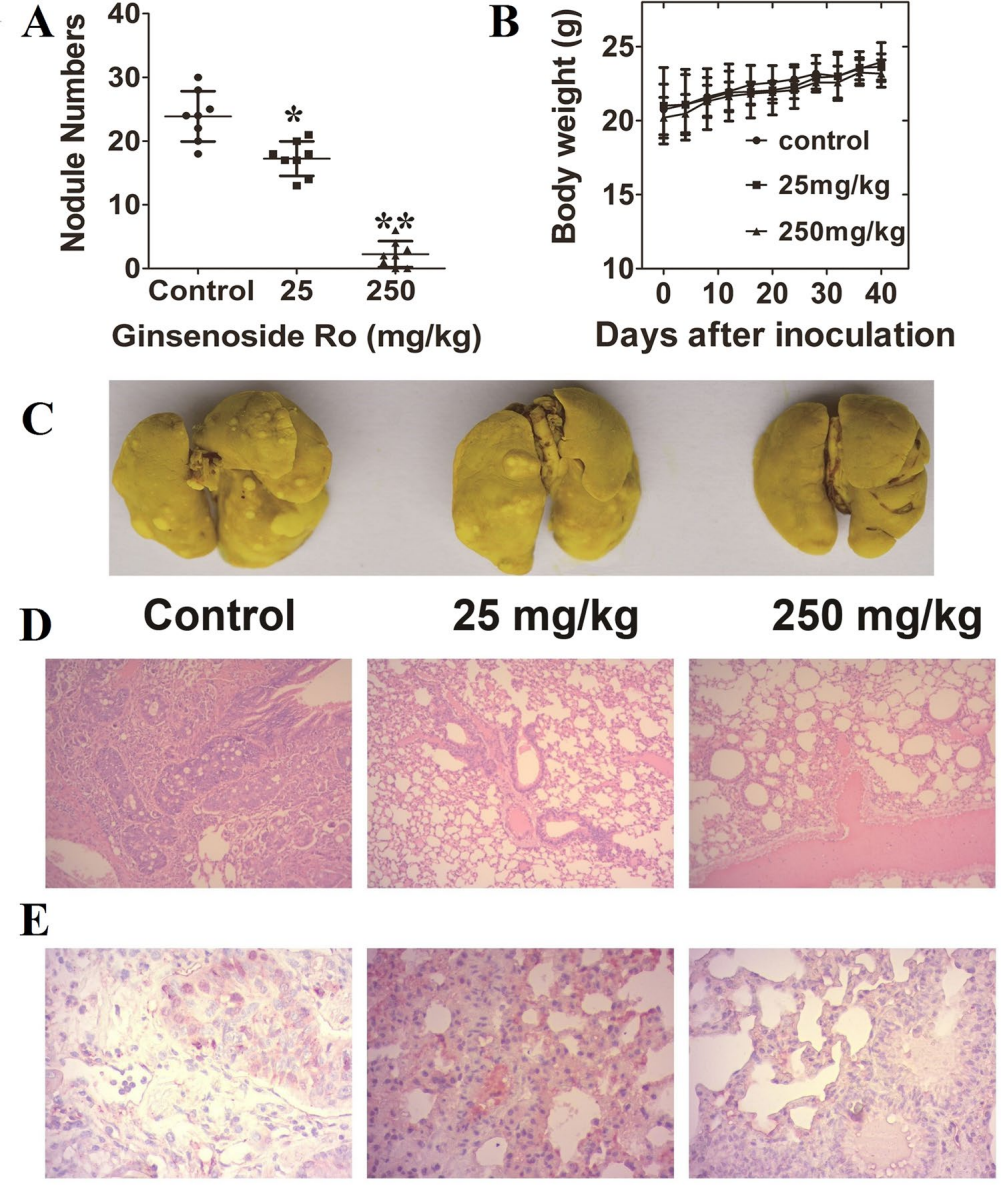
Effect of oral ginsenoside Ro on lung metastasis induced by HT29 cells tail-injected into female Balb/c nude mice. (**A** and **B**) Four-day pretreatment with ginsenoside Ro before HT29 injection followed by the same treatment for 40 days significantly reduced the number of tumor nodules on the mouse lung surface without affecting the mouse body weight in comparison with the untreated control. (**C**) Representative images of the lungs taken from different treatment groups. (**D**) H&E staining of the paraffin-embedded lungs showed a decrease in metastatic foci by ginsenoside Ro dose-dependently. (**E**) Immunohistochemical staining showed the expression of integrin αvβ6 in mouse lungs was reduced by ginsenoside Ro dose-dependently. Data represent the mean ± SD (n = 10 per group). *P < 0.05, and **P < 0.01, compared with the control.

The above *in vitro* experiments tempted us to further examine whether ginsenoside Ro could inhibit or prevent the intravenous HT29 from metastasis to lungs of nude mice. Ginsenoside Ro dissolved in water was administrated by gavage to mice at doses of 25 and 250 mg/kg/day for 4 days before *i.v.* injection of HT29 in order to keep blood concentrations of ginsenoside Ro above a certain level before HT29 *i.v*. injection followed by 40 days of oral administration of ginsenoside Ro to the mice. After 38 days of treatment, the animals were euthanized, and the number of pulmonary metastatic nodules was counted in addition to evaluation of toxicity of ginsenoside Ro and mouse pathology by HT29. Ginsenoside Ro (250 mg/kg/day) produced a significant decrease in the number of tumor nodules on the lung surface, yielding inhibition rates of 88% (*P* < 0.01; Fig. 6A,C). However, there was no significant difference in body weight between the control and treated groups (Fig. 6B). Histopathological H&E staining of various lung sections revealed that ginsenoside Ro produced significant decrease in HT29-induced metastatic lesions and tissue density in comparison with that in the vehicle-treated group. The results demonstrated that ginsenoside Ro-mediated reduction in lung metastasis was significant, and the ginsenoside Ro-treated mice showed no toxic signs throughout the experiments. In addition,Lungs from the three groups (control, low dose and high dose) were stained with the integrin *α*v*β*6 antibody. Representative pictures are shown in Fig. 6E, in which the red-brown color indicates immunoreaction decreased when treated dose was increased.

## Discussion

In the present study, we discovered, for the first time, that all 5 compounds separated and purified from the well-known abortion TCM *A. bedentata* possessed good capacity in inhibiting implantation of human embryo to human endometrium. Among them, ginsenoside Ro appeared to be the most potent inhibitor (Fig. 1). We provided, for the first time, the cellular evidence to support *A. bedentata* as the abortion TCM, and its cellular mechanism of action as the abortion TCM (Fig. 2). Previous researches on ginsenoside Ro mostly involved in the anti-inflammatory^28, 29^, thrombosis prevention^30, 31^ and anti-oxidation^32^. The most eye-catching newly discovery of ginsenoside Ro is its autophagy inhibition activity, which beyond several other ginsenosides extraceted form Panax ginseng^33^. Herein we demonstrated that ginsenoside Ro inhibited migration and invasion ability of cancer cells and their adhesion to human endothelial cells (Fig. 4) via its specific inhibition of expression of integrin *αvβ*6, MMP-2 and MMP-9 (Fig. 5) without producing any significant cytotoxicity to the tested cells (Fig. 3). The novel pharmacological mechanisms of action of ginsenoside Ro were further supported by the *in vivo* study results that demonstrated that ginsenoside Ro could safely and effectively prevent circulating tumor cells from metastasizing to mouse lungs and downregulated tumor integrin *αvβ*6 expression (Fig. 6).

Human embryo implantation is a critical multistep process consisting of embryo apposition-adhesion, followed by penetration and invasion. Through embryo penetration, the endometrial epithelial cell barrier is disrupted and remodeled. Uchida *et al*. previously developed an *in vitro* model for human embryo implantation employing the human choriocarcinoma cell line JAG-3 and the human endometrial adenocarcinoma cell line RL95-2^34^. We demonstrated that ginsenoside Ro and other compounds interrupted the implantation between JAG-3 and RL95-2. We recently found many molecular similarities between the embryo implantation system and CTC adhesion system. The two systems share the high expression of sLex, CD47, Ep-CAM, integrin *α*5, *α*6, *β*1, and other adhesion molecules. This finding inspired us to *A. bedentata* as a cancer metastatic chemopreventive since it is the TCM for abortion, and possesses a good safety profile (Fig. 6), the leaf methanol extract of *A. aspera* was reported no toxicity to mice at 2000 mg/kg (p.o.)^35^.

Metastatic spread is a complex process initiated by the dissemination, seeding and engraftment of CTCs to the distant metastatic tissues. The series of consequential events include the activation of dormant CTCs, adhesion between CTCs and vascular endothelial bed of distant organs, and the continued survival and initial proliferation of CTCs after extravasation. We have proposed that activation-adhesion of CTCs to the vascular bed is a crucial starting point of the metastatic cascade for chemo-intervention^36^. If we can control the starting point, we may effectively prevent cancer metastasis. There are fundamental differences among chemotherapy, cancer chemoprevention and cancer metastatic chemoprevention in terms of mechanisms of action and biomarkers^9^. We defined cancer metastatic chemoprevention as comprehensively preventing the activation-adhesion-extravasation-proliferation metastatic cascade sparked by CTCs. In particular, our cancer metastatic chemoprevention strategy focuses on preventing the initial step of the cancer metastatic cascade following removal of primary tumors.

The microenvironment surrounding CTCs plays an important modulating role in CTC-based cancer metastasis and must have a decisive effect on whether the CTCs could seed (or adhere) in the soil (the single layer of the flat endothelium). A large number of interconnected factors have individual impact on the complex CTCs activation-adhesion process. They include: perfect spatiotemporal fitting (such as vascular tension, blood flow shear stress), the activities of inflammatory factors and the amount of cellular adhesion molecules (CAMs), selectins, platelets and integrins. The present study focused on the role of integrins in cancer metastasis, and found that the ginsenoside Ro extracted from *A. bidentata* is the most potent among the five compounds in inhibiting embryonic implantation to endometrium and adhesion of cancer cells to endothelial HUVECs within the non-cytotoxic concentration range. The molecular basis for the inhibition of the hetero-cellular adhesion, migration and invasion seems to be related to the integrin *αvβ*6 (Figs 4 and 5).

CTCs are integrin-dependent adhesion to ECM components in comparison to non-transformed cells^37, 38^. Within the integrin subfamily, *αvβ*6 is not expressed in normal epithelia. However, it becomes highly expressed during tumorigenesis and the *β*6 integrin subunit is thought to be widespread in cancers of the lung, breast, pancreas, ovary, oropharynx and colon^39, 40^. Heterologous expression of *αvβ*6 in a colon cancer cell line has been shown to be related, in part, to *αvβ*6-mediated MMP-9 secretion^25^. We studied the effects of ginsenoside Ro on integrins including *β*1, *α*6 and the *αvβ*5, *αvβ*6 and E-cadherin by flow cytometric analyses (Fig. 5B), and found that only *αvβ*6 expression was down-regulated significantly by ginsenoside Ro. Also, immunohistochemical staining of lung tissue with an integrin *αvβ*6 showed Integrin *αvβ*6 expression in treated mouse lung decreased with the increasing ginsenoside Ro dose (Fig. 6E).

MMPs are the key regulatory enzymes for infiltrating into the surrounding tissue through degradation of basal membranes and extracellular matrix, which is one of the critical steps in the cascade of metastasis^41, 42^. The expression of MMP-2 and -9 is higher in colon tumor tissues than in normal tissues. The breakdown products and growth factors released by the MMPs can stimulate migration and enhance the invasive behavior of the cancer cells^43^. Without such enzymatic activity, cancer cells would probably be unable to traverse barriers such as basement membrane or stromal extracellular matrix. Our present work revealed that ginsenoside Ro reduced the expression of MMP-2 and -9 and relative transcription in a concentration-dependent manner. Furthermore, it was reported that higher expression of p-ERK1/2 was associated with integrin *αvβ*6 expression^25^. When non-phosphorylated ERK binds *β*6, ERK can be more efficiently phosphorylated by MEK because of conformational changes. Therefore, ERK signaling was critical for *β*6-induced MMP-2 and -9 expressions.

## Conclusions

**Figure 7:**
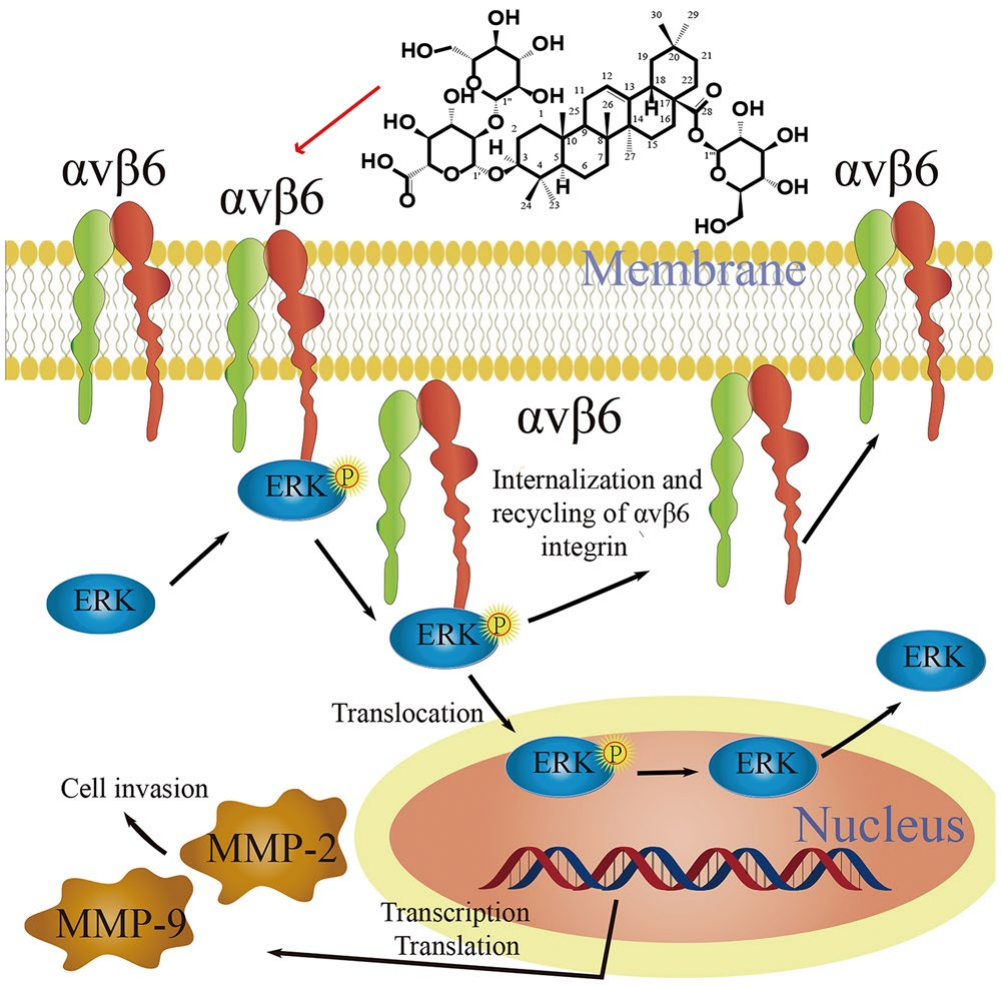
The possible mechanism underlying the inhibition by ginsenoside Ro of cancer cell invasion and metastasis. Ginsenoside Ro inhibits integrin αvβ6 and phosphorylation of ERK, which further results in inhibition of the formation of integrin β6/ ERK complex intracellularly. The inhibition of ERK phosphorylation somehow reduces the expression of MMP-2 and MMP-9. Together, the adhesion, invasion and metastatic ability of cancer cells are reduced.

Our present study revealed that the expression of p-ERK1/2 in HT29 cells was decreased by ginsenoside Ro. The inhibited expression by ginsenoside Ro of *αvβ*6 may result in the blockage of the direct binding between *β*6 and ERK (Fig. 7), leading to the reduced p-ERK1/2 expression and alleviated ability of HT29 to adhere, migrate and invade.

## Methods

### Reagents and antibodies

Human tumor necrosis factor alpha (TNF-*α*), E-cadherin and mouse anti-human *αvβ*5, *αvβ*6, *α*6, *β*1, GAPDH antibody were purchased from Cell Signaling Technology Inc. The primary antibodies anti-MMP-2, anti-MMP-9, anti-ERK, anti-pERK were obtained from Abcam. The secondary antibody goat anti-mouse-IgG horseradish peroxidase was obtained from Promega. Fibronectin was obtained from Sigma-Aldrich. Basement Membrane Matirx (Matrigel™) was purchased from BD.

### Plant material and Separation of crude elution

Fresh roots of the plant *A. bidentata* were collected from AnHui China. It was authenticated by plant taxonomist Zehao Huang Ph.D., Pharmaceutical College, Fujian University of Traditional Chinese Medicine, China. Dried *A. bidentata* roots were cut into pieces and extracted with 80% refluxing ethanol. The extraction was partitioned with petroleum ether to remove fatty substances, and n-Bu-OH to get the middle polarity compositions, respectively. And the residue from the n-Bu-OH was subjected to a porous polymer resin D101 column eluting with water/ethanol at 100:0, 7:3 and 1:4 for collecting. 80% ethanolic elution was remained.

### Phytochemical analysis

80% ethanolic elution was subjected to qualitative tests for the identification of various active constituents’ viz. carbohydrate, glycoside, alkaloid, saponin, phytosterols etc. according to Kokate CK^44^ and KhandelwalKR^45^. Molisch’s test and Fehling’s test for carbohydrates and glycosides; Salkowski reaction, Libermann-Burchard’s test and Foam test for phytosterols and saponins; Mayer’s reagent, Dragendroff’s reagent, Hager’s test and Wagner’s test for Alkaloids.

The fractionation of the crude elution was performed on a semi-preparative C18 column (19 × 150 mm, 5 µm) with a Waters HPLC system that consisted of a Waters 2695 HPLC pump and a Waters 2480 UV-Vis detector. The mobile phases were composed of CH3CN (phase B) and 0.1% formic acid aqueous (phase A), with an isocratic of 15% B in 45 min and a gradient of 31.5% B to 34% B in 25 min, to 40% B in 15 min and then to 67.5% B in 10 min. The flow rate was 10 ml/min, and the column temperature was maintained at 35 °C. The amount of sample loading is 100 mg per injection.

Structure elucidation of isolated active compounds was carried out by using spectral techniques. ESI-MS were recorded on a Q-TOF system (Waters). NMR spectra were obtained on an AVANCE III 500 MHz NMR system (Bruker, Switzerland).

### Cell culture

HT29 colorectal carcinoma cell line, JEG-3 human choriocarcinoma cell line and RL95-2 human uterine endometrial carcinoma cell line were purchased from the Chinese Academy of Sciences Cell Bank of Type Culture Collection (Shanghai, China). HT29 cells were cultured using McCoy’s 5 A medium; JEG-3 cells were cultured in DMEM medium, and RL95-2 cells were cultured in RPMI-1640 medium supplemented with 10% fetal bovine serum (FBS), 100 *μ*g/mL streptomycin and 100 U/mL penicillin in a 5% CO_2_ and humidified atmosphere at 37 °C. Human umbilical vein endothelial cells (HUVECs) were isolated as described before^36^, and cultured on gelatin-coated culture dishes in M199 medium supplemented with 20% heat-inactivated fetal bovine serum, penicillin (100 U/mL), streptomycin (100 *μ*g/mL), and endothelial cell growth supplement (ECGS) (BD BiocoatTM) at 37 °C and 5% CO_2_. HUVEC between P3 and P5 were used for all experiments.

### Mice

Female BALB/c mice (20–25 g, 6–8 weeks old) were obtained from Shanghai SLAC Laboratory. These mice were housed in a separate room, and maintained with free access to pellet food and water at 20–25 °C, in 12-h light/ dark circles and under 50–60% relative humidity condition. According to the Guide for the Care and Use of Laboratory Animals^46^, mice used in the investigation were handled, and approved by the institutional animal care and use committee of Fuzhou University.

### Spheroid Attachment Assay

Human endometrial epithelial cells (RL95-2) were seeded into 96-well plates, 3 × 10^5^ per well. And confluent monolayer was formed after incubation for 24 h. Trophoblast spheroids were generated by cell aggregation on a fast-rocking shaker at 90 rpm for 24 h. At the end of spheroid preparation, the spheroid suspension was labeled with Calcein-AM fluorescent dye and incubated for the left 30 min. Spheroids (size = 70–100 *μ*m) were added on the top of RL95-2 monolayers and were incubated for 1 h. Attached spheroids were counted by fluorescent microscope and the percentage of attachment was calculated as number of attached spheroids/number of seeded spheroids.

### Cytotoxic assay

To determine the cytotoxicity of ginsenoside Ro, the 3-(4, 5-dimethylthiazol-2-yl)-2, 5-diphenyltetrazolium bromide (MTT) assay was used. Details of procedure are as previously described^47^. After overnight incubation, different concentrations (1–100 *μ*g/mL) of ginsenoside Ro were added into triplicate wells. The corresponding cytotoxicity values were calculated (*λ* = 554 nm). Each test was repeated at least three times.

### Apoptosis

Following the treatment time course, annexin V (Strong Biotect Corporation, Taipei, Taiwan)^48^/PI (Sigma, St Louis, MO, USA) method was employed^49^. Cells were washed, trypsinized, suspended in binding buffer and stained with recombinant fluorescein isothiocyanate (FITC)-conjugated Annexin-V and PI. After supravital staining, the analysis of flow cytometer was performed immediately.

### Migration assay

A wound-healing migration assay was performed using the established protocol^24^. HT 29 cells were seeded in a six-well plate at the destiny of 5 × 10^5^ cells per well, which were pre-coated with fibronectin (5 *μ*g/mL; Sigma-Aldrich), to form a monolayer. Subsequently, a scratch was made and washed with starvation medium to remove detached cells. Ginsenoside Ro was added to the serum-free medium at the concentration of 20 and 50 μg/mL. At indicated time-points, images of each well were captured. The migration ability was compared by measuring the width of marked area as the distance that cells migrated.

### Invasion assay

To analyze the effect of ginsenoside Ro on the invasion of HT29 cell line, a transwell invasion assay was carried out as previously described^50^. Briefly, the upper culture compartments fitted with polyethylene terephthalate (8 *µ*m pore size) in 24-well dishes were pre-coated with collagen matrix (80 *μ*L of serum-free RPMI 1640-diluted matrigel). Overnight, 5 × 10^4^ cells resuspended in 200 *μ*L of serum-free McCoy’s 5 A medium containing ginsenoside Ro at indicated concentrations (20 and 50 *μ*g/mL) are placed into the upper compartments of wells, and 600 *μ*L McCoy’s 5 A medium supplemented with 20% FBS was added to the lower compartment of each well to serve as chemoattractant. Three duplicate wells are set up for each group. After 24 hours of incubation at 37 °C and 5% CO_2_, the cells that did not penetrate through the membrane were wiped out carefully by a cotton wool, and the filters were fixed with methanol for 30 minutes and then dyed with crystal violet. Cell invasion ability was determined from the number of cells penetrating through the membrane.

### Adhesion assay

The cell adhesion assay was performed as previously described^47^. The tissue culture plates of 96-well were coated with 2 *μ*g of matrigel and was dried in a laminar flow cabinet overnight at room temperature. After washing three times with PBS to remove excess and unbound Matrigel, the wells were blocked with 20 mL of a 20 mg/L bovine serum albumin (BSA, Sigma) solution in McCoy’ 5a medium for 1 h at 37 °C. At a density of 8 × 10^5^ cells/mL, cells were suspended in serum-free McCoy’s 5 A medium containing ginsenoside Ro at indicated concentrations (20, 50 and 100 *μ*g/mL), and 100 *µ*L of the cell suspension was added to each well for 1 h of incubation at 37 °C. Then, the wells were washed with PBS 3 times to remove the non-adherent cells. Finally, MTT solution was added, and incubated for measurement with microplate reader, as we described previously^51^. The adhering rate was calculated as this: (A_570nm_ of the treated cells- A_570nm_ of background)/ (A_570nm_ of the control group- A_570nm_ of background).

Human umbilical vein endothelial cells (HUVECs) were collected as previously described^52^. They were cultured in 24-well plate pre-coated with 1% gelatin until grown to confluence. HT29 cells (10^5^ cells per well) labeled with rhodamine 123 were plated in a final volume of 500 *µ*L M199 medium containing indicated concentration of ginsenoside Ro on the HUVECs monolayer untreated or stimulated with 10 *μ*M TNF-*α* for 4 h.

After incubation, wells were washed 3 times with PBS to remove unattached HT29 cells. Pictures of randomly selected visual fields for each well were taken under a fluorescence microscope (Zeiss, Germany). Adherent cells were counted by using ImageJ software, and data are presented as inhibition percentage versus the control value.

### Flow cytometry

Flow cytometer (BD FACSAriaIII) was employed to detect cells expression of integrin *αvβ*5, *αvβ*6, *α*6, *β*1, and E-cadherin, as we described previously^53^. Following the treatment time course, HT29 cells were washed twice in PBS and stained with antibodies at 4 °C for 20 min. Subsequently, secondary conjugated antibody was added for visualization in flow cytometer. Data acquisition and analysis were performed using BD FACSDiva software.

### Western blot analysis

HT29 cells were treated with ginsenoside Ro at various concentrations (0, 20, 50, 100 *μ*g/ml) for overnight incubation. Subsequently, cells were rinsed twice with cold phosphate-buffered saline and then lysed with lysis buffer (100 *μ*L of RIPA buffer containing 1 mM PMSF). The cell lysates were configured at 12000 g for 5 min to isolate protein lysate. Using a microbicinchoninic acid protein assay (BCA), the concentrations of protein were determined for western blot analysis, as previously described^7^. The concentration of samples was measured by BCA Protein Assay Kit. Equal amounts of protein (20 *μ*g/well) were separated bysodium dodecylsulfate-polyacrylamide gel electrophoresis (SDS-PAGE, Bio-Rad) and transferred in a polyvinylidene difluoride (PVDF, Bio-Rad) membrane. The membrane was blocked in 5% skim milk for 1 h at room temperature, and then probed with primary antibodies overnight at 4 °C and an additional 1 h incubation with the appropriate HRP-labeled secondary antibody. The dilutions of the primary antibodies were all 1:1000 and that of the secondaryantibody was 1:5000. The target protein expression was detected by enhanced chemiluminescence (ECL Kit) and quantified with Bio-Rad Quantity One software analysis system, with normalization to GAPDH levels.

### Quantitive real-time PCR

Total RNA was isolated from HT29 cells as described by Chomczynski and Sacchi^54^. Subsequently, cDNA was synthesized from 10 *μ*g purified RNA, and PCR reactions were performed using the SYBR Green real-time PCR method on the CFX96™ Real-Time PCR Detection Systems (Bio-Rad). The following sequences of gene-specific primers were:

*αvβ*6: F 5′-AGAACTCTAAGCAA-3′; R 5′-AAAGTTGGTGGAACCTCG-3′; MMP-2: F 5′-AAGTCTGAAGAGCGTGAAG-3′; R 5′-CAGGTAGGAGTGAGAAGC-3′; MMP-9: F 5′-TGACAGCGACAAGAAGTG-3′; R 5′-CAGTGAAGCGGTACATAGG-3′; GAPDH: F 5′-CGGAGTCAACGGATTGGTGTT-3′; R 5′-AGCCTTCTCCATGGTTGGTGA AGAC-3′.The transcript levels of target mRNA were normalized with the GAPDH mRNA using the 2^−∆∆CT^ method.

### Development of lung metastasis induced by HT29 cells

The experimental model of lung metastasis was established by tail vein injection of HT29 cells to mimic the dissemination of CTCs. HT29 cells in the number of 2 × 10^6^ cells in 0.2 ml PBS were infected into the tail vein of six-week-old female Balb/c mice. Before the HT29 inoculation, oral gavage pretreatment of PBS-suspended B (ginsenoside Ro) was given daily for 4 days, followed by a 40-day treatment. Treatment groups (N = 10) included: 0 mg/kg, 25 mg/kg and 250 mg/kg ginsenoside Ro. Body weight was measured and recorded every four days. Mice were sacrificed after 40 days of tumor metastasis and growth and 44 days of treatment with B. The number of surface lung metastasis nodules was evaluated in each treatment group. Slides with 4–5 μm thick lung section were prepared, paraffin embedded and then stained with hematoxylin and eosin (H&E).

### Immunohistochemistry

For immunohistochemical analysis, the paraffin embedded tissues were cut into 6 *μ*m sections and stained with anti-$\alpha v\beta 6$ antibody as described elsewhere^55^. Integrin $\alpha v\beta 6$ expression in the lung tissue was performed using avidin-biotin complex (ABC) method. Stained cells were visualized under light microscope at ×200 magnification.

### Statistical analysis

Data represents the means ± S.D. in each experiment. One-way analysis of variance was employed to performed statistical analysis. The SPSS statistical software (version 19.0) was used to determine the statistical significance between the means. P-values < 0.05 were considered statistically significant, and *P* < 0.01 to be highly statistically significant.

## Electronic supplementary material

Supplementary information
