# Supplementary material for: The traditional Chinese medicine Achyranthes bidentata and our de novo conception of its metastatic chemoprevention: from phytochemistry to pharmacology
Source: Sci Rep. 2017 Jun 20;7:3888. doi: 10.1038/s41598-017-02054-y (PMC5478643; doi:10.1038/s41598-017-02054-y)
Supplement: Supplementary file 1 — Supplementary information [file 41598_2017_2054_MOESM1_ESM.doc]

**The traditional Chinese medicine *Achyranthes bidentata* and our *de novo* conception of its metastatic chemoprevention:**

**from phytochemistry to pharmacology**

Zhou Jiang^1,3^, Jun Qian^1,3^, Haiyan Dong^1,2,3^, Jingyi Yang^1^, Xiaobo Yu^1^, Jianzhong Chen^1^, Hongning Chen^1^, Qing Shi^1^, Lee Jia^1^,*

^1^ Cancer Metastasis Alert and Prevention Center, and Pharmaceutical Photocatalysis of State Key Laboratory of Photocatalysis on Energy and Environment, College of Chemistry; Fujian Provincial Key Laboratory of Cancer Metastasis Chemoprevention and Chemotherapy, Fuzhou University, Fuzhou 350002, China.

^2^ Rutgers, The State University of New Jersey,160 Frelinghuysen Road, Piscataway, NJ 08854-8020, USA.

^3^ Co-first author.

* Correspondence: [pharmlink@gmail.com](mailto:pharmlink@gmail.com),[cmapcjia1234@163.com](mailto:cmapcjia1234@163.com) (L.J.)

**Supplementary Figure S1**. ESI-MS data for compound 1 to 3.


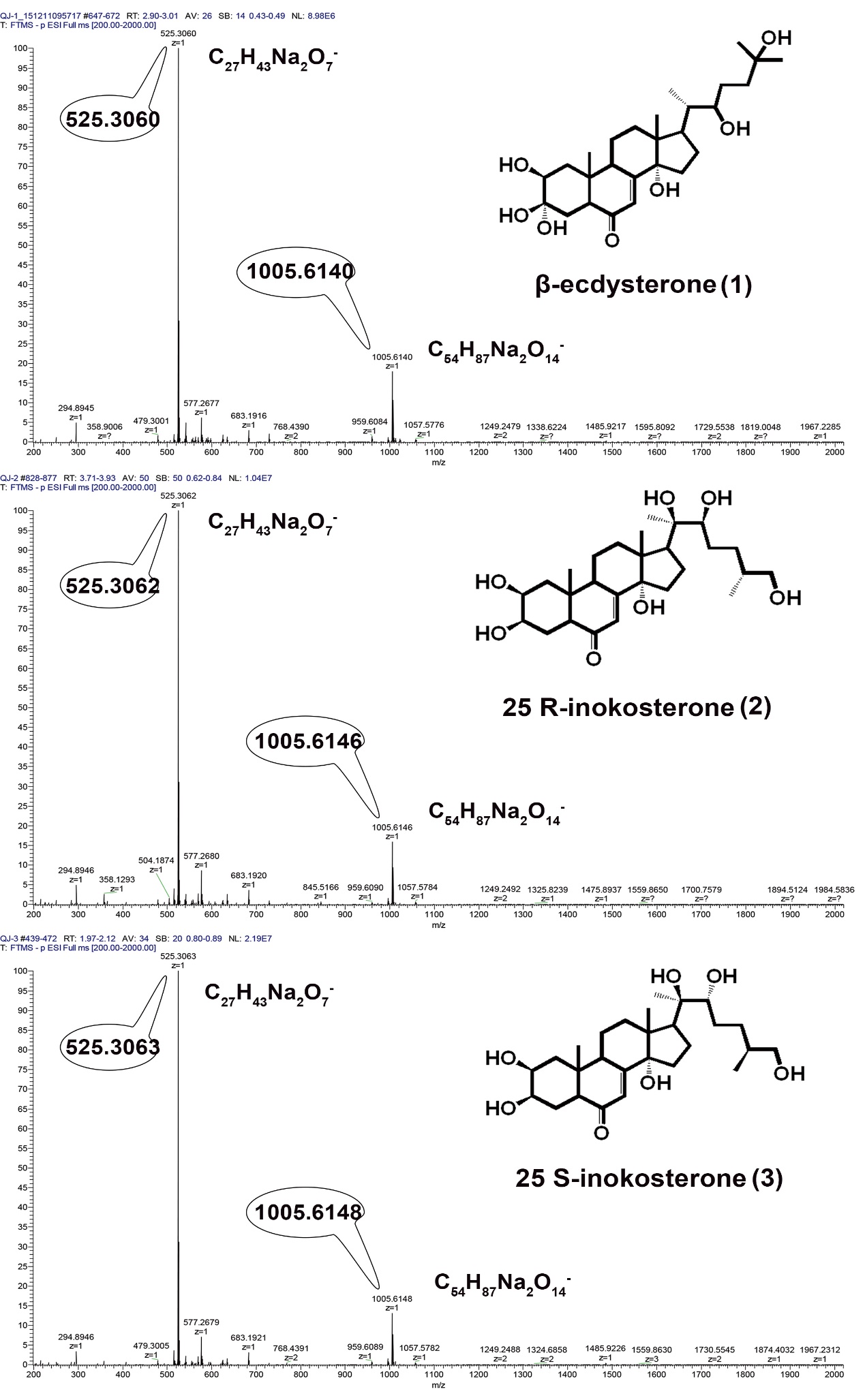


**Supplementary Figure S2**. ESI-MS data for compound B.


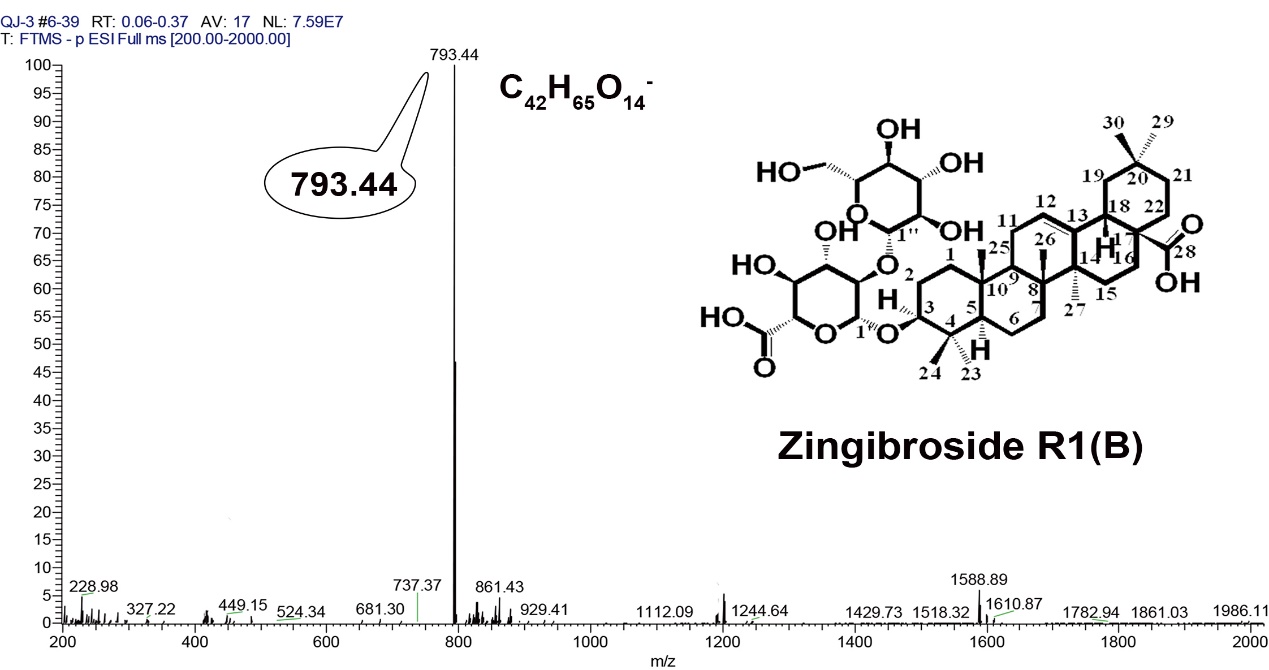


**Supplementary Table S1**. ^13^C-NMR spectral data for ginsenoside Ro (500MHz, C5D5N-d_5_, δ, ppm)

| Compound | Ginsenoside Ro | | |
| --- | --- | --- | --- |
| Formula | C_48_H_75_O_19_ | | |
| Structure |  | | |
| Position | δC | Position | δC |
| 1 | 38.4 | 27 | 25.8 |
| 2 | 26.3 | 28 | 176.1 |
| 3 | 88.9 | 29 | 32.3 |
| 4 | 39.6 | 30 | 23.4 |
| 5 | 55.5 | -*O*-Glc |  |
| 6 | 18.2 | 1΄ | 105.0 |
| 7 | 32.8 | 2΄ | 82.5 |
| 8 | 39.2 | 3΄ | 76.8 |
| 9 | 47.7 | 4΄ | 72.8 |
| 10 | 36.6 | 5΄ | 77.4 |
| 11 | 23.3 | 6΄ | 172.1 |
| 12 | 122.9 | 1΄΄ | 105.7 |
| 13 | 143.8 | 2΄΄ | 77.6 |
| 14 | 41.8 | 3΄΄ | 77.9 |
| 15 | 28.0 | 4΄΄ | 70.8 |
| 16 | 23.1 | 5΄΄ | 77.2 |
| 17 | 45.9 | 6΄΄ | 62.4 |
| 18 | 41.4 | 1΄΄΄ | 95.4 |
| 19 | 46.7 | 2΄΄΄ | 73.8 |
| 20 | 30.5 | 3΄΄΄ | 79.0 |
| 21 | 33.7 | 4΄΄΄ | 71.4 |
| 22 | 32.8 | 5΄΄΄ | 78.6 |
| 23 | 27.8 | 6΄΄΄ | 61.9 |
| 24 | 16.4 |  |  |
| 25 | 15.2 |  |  |
| 26 | 17.2 |  |  |
